# The neural niche in cancer: mechanistic insights into tumor–neuron–immune crosstalk and therapeutic opportunities

**DOI:** 10.3389/fcell.2026.1667459

**Published:** 2026-03-10

**Authors:** Nese Aysit, Esra Altintas, Fulya Koksalar Alkan, Gurkan Ozturk, Hasan Korkaya

**Affiliations:** 1 Department of Medical Biology, School of Medicine, Istanbul Medipol University, Istanbul, Türkiye; 2 Research Institute for Health Science and Technologies (SABITA), Istanbul Medipol University, Istanbul, Türkiye; 3 Karmanos Cancer Institute, Department of Oncology, Wayne State University School of Medicine, Detroit, MI, United States; 4 Department of Physiology, Bolu Abant Izzet Baysal University School of Medicine, Bolu, Türkiye

**Keywords:** adrenergic signaling, cancer-neuron-immune axis, neurotransmitters, neurotrophic factors, perineural invasion, tumor innervation, tumor microenvironment

## Abstract

The nervous system is increasingly recognized as a dynamic and regulatory component of the tumor microenvironment playing critical roles in cancer initiation, progression, metastasis, and resistance to therapy. Recent evidence in cancer neuroscience have revealed a specialized “neural niche” a microanatomical and functional domain enriched in neural inputs and neuromodulatory signals orchestrated through bidirectional communication between tumor, nervus system and immune cellsCancer cells secrete neurotrophic factors such as nerve growth factor (NGF), brain-derived neurotrophic factor (BDNF), and glial cell line-derived neurotrophic factor (GDNF) to attract and remodel peripheral innervation. Infiltrating nerve fibers, in turn, release neurotransmitters (e.g., norepinephrine, acetylcholine) and neuropeptides (e.g., substance P, calcitonin gene-related peptide) that influence not only tumor growth, angiogenesis but also immune cell polarization, T cell exhaustion, dendritic cell maturation and myeloid derived suppressor cell recruitment. This neural-immune crosstalk establishes immune suppressive microenvironment that facilitates tumor immune escape and leading to metastatic progression. Perineural invasion (PNI), a distinct pathological process of tumor dissemination, further exemplifies neuroepithelial integration and correlates with recurrence, pain and poor prognosis across multiple solid tumors. Beyond local interactions, chronic stress and systemic neuroendocrine activation via the hypothalamic-pituitary-adrenal (HPA) axis and sympathetic-adrenal-medullary networks, contribute to tumor-promoting immunosuppression through glucocorticoid signaling and sympathetic responses. In this review, we discuss mechanistically integrated and clinical relevant synthesis of tumor-neuron-immune interactions. We emphasize recent conceptual advances, including autonomic balance, systemic neuroendocrine feedback and therapeutic strategies targeting this axis. These insights establish a framework for future translational research and development of neuromodulatory therapies that complement immunotherapy as well as conventional therapeutics.

## Introduction

Cancer develops in a dynamic tumor microenvironment (TME) composed of diverse cell types and signaling molecules that collectively influence tumor growth and metastasis (de Visser and Joyce, 2023). The TME consists of immune cells, fibroblasts, endothelial cells, adipocytes, extracellular matrix and soluble factors (cytokines, chemokines and growth factors) which collectively drive hallmarks of cancer such as sustained proliferation, angiogenesis, invasion, and immune evasion (Hanahan, 2022). Over the past decade, accumulating evidence suggested that the nervous system is also an integral part of the TME playing a critical role in driving aggressive properties of the cancer (Wang et al., 2021). This emerging paradigm has given rise to the field of “cancer neuroscience” a multidisciplinary area focusing on the intricate interactions between the nervous system and cancer. *Cancer neuroscience* conceptualizes tumors as tissues fully integrated into neural circuits that malignant cells not only receive neural signals but can also actively modulate neural structure and function (Hwang et al., 2025). Cancer neuroscience examines how neural inputs (from both central and peripheral nervous systems) can influence tumor initiation, growth, immune evasion, and metastasis, and conversely how tumors can alter neuronal pathways locally and systemically. By integrating neuroscience with oncology, this framework anchors our understanding of phenomena such as stress-related tumor progression and cancer-induced neural remodeling within a cohesive conceptual field (Wang et al., 2021; Chen et al., 2019).

While prior reviews have explored aspects of neural involvement in cancer biology, our review discusses a unified framework that connects local tumor-nerve-immune interactions with systemic neuroendocrine modulation, integrating recent advances across cancer neuroscience, immunology, and endocrinology. We define and delineate the concept of a “neural niche,” highlighting its structural, signaling, and immunological components. This review aims to bridge gaps between preclinical models and translational opportunities in targeting the cancer-neuro-immune axis.

The nerve fibers have been identified as one of the players in TME in a variety of cancers, including pancreatic, colorectal, prostate, breast, and head and neck tumors (Wang et al., 2021). Tumors were long thought to spread predominantly via vasculature and lymphatics; however, the discovery of tumor cells migrating along nerves and the outgrowth of neurites into tumor tissue has revealed an underappreciated route of dissemination called “perineural invasion” in head and neck tumor (Brown et al., 1989). This bidirectional interaction between cancer and the nervous system, sometimes termed *cancer neuroscience*, is now a growing field of research (Wang et al., 2021). Tumors can stimulate neurite outgrowth (neo-neurogenesis) and nerve plasticity in their vicinity, while neural inputs (both central and peripheral) can regulate tumor biology at local and systemic levels (Lolas et al., 2016; Wang et al., 2024a; Silverman et al., 2021).

In this review, we define the neural niche as a spatial and functional network within the TME enriched with infiltrating nerve fibers, neurotrophic factors, neurotransmitters and neuropeptides, along with immune and stromal mediators responsive to neural signals (Sloan and Lee, 2025). Unlike the immune or stromal architecture, which are primarily organized around immune modulation and structural support, neural niche is characterized by neuro-epithelial-immune crosstalk that actively influences tumor progression, immune evasion and neural remodeling. This niche is shaped not only by local innervation but also by systemic neural inputs such as those from the hypothalamic-pituitary-adrenal (HPA) axis. Furthermore, key processes such as perineural invasion (PNI), neural guidance of metastasis, and formation of immunosuppressive microenvironment are examined. We also highlight systemic neuro-immune networks, such as stress-activated neuroendocrine pathways regulating tumor growth and progression beyond the local microenvironment (Zhang et al., 2025a).

Finally, we critically examine limitations in current models and experimental approaches, including species-specific differences, context-dependent autonomic effects, and tumor heterogeneity. Recognizing these challenges, we propose future research directions and therapeutic strategies that leverage neuromodulation to disrupt tumor-supportive neural circuits improving the efficacy of immunotherapies.

### Tumor microenvironment

**FIGURE 1:**
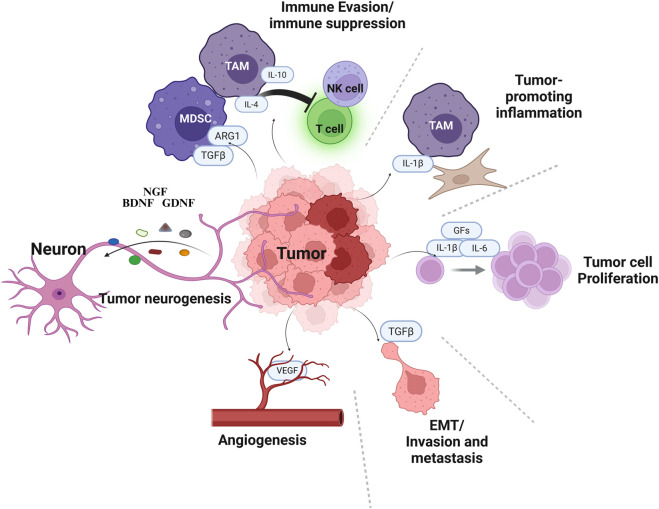
Neuronal regulation of cancer hallmarks. The complex cellular architecture of the tumor microenvironment (TME), highlighting interactions among cancer cells, immune cells, and neurons. TME is composed of differentiated cancer cells and cancer stem cells (dark red), surrounded by diverse cell types including endothelial cells (vasculature), fibroblasts, tumor associated macrophages (TAMs), myeloid derived suppressor cells (MDSCs), T cells and NK cells. Neurons recruited by the tumor cells play a major role in generation of the immune suppressive and inflammatory microenvironment. Created in BioRender. Korkaya, H. (2026) https://BioRender.com/r4qfx04.

The tumor microenvironment (TME) is a dynamic and complex ecosystem composed of malignant cells, stromal elements, immune infiltrates, vasculature, and increasingly recognized neural components. These diverse cellular and molecular constituents engage in reciprocal interactions that shape tumor behavior, immune responses, and therapeutic outcomes. Within this evolving niche, nerve fibers and neuroactive signals integrate with immune and stromal pathways to modulate cancer progression, highlighting the TME as both a structural and signaling hub for tumor-neuron-immune crosstalk. Immune cells in the TME can be co-opted by tumors: for example, tumor associated macrophages and lymphocytes may secrete growth factors, remodel the extracellular matrix, and create immunosuppressive conditions which favors tumor growth and dissemination (de Visser and Joyce, 2023; Binnewies et al., 2018). Cancer-associated fibroblasts (CAFs) (Yang et al., 2023; Sahai et al., 2020), other stromal cells as well as the tumor cells produce extracellular matrix components and pro-tumorigenic signals that facilitate invasion and metastasis. Endothelial cells form blood vessels that provide nutrients for the growing tumors (Yao and Zeng, 2023; Akino et al., 2009). Immune suppressive myeloid cell populations such as the tumor associated macrophages (TAMs) or myeloid derived suppressor cells (MDSCs) suppress the anti-tumor immune responses (Cassetta and Pollard, 2023; Ouzounova et al., 2017; Bronte et al., 2016). Collectively, these interactions were conceptualized in the Hallmarks of Cancer, which include sustained proliferative signaling, resistance to cell death, angiogenesis, invasion, and metastasis (de Visser and Joyce, 2023; Hanahan, 2022) (Figure 1).

## Tumor-immune-neuron interactions

While cancer-associated fibroblasts (CAFs), tumor associated macrophages (TAMs), myeloid derived suppressor cells (MDSCs), and endothelial cells have been widely studied, the role of the nervous system in the tumor microenvironment (TME) has only recently emerged as a key area of investigationInnervation is particularly prominent in pancreatic, head and neck, gastric, and prostate cancers, where neural fibers infiltrate tumor tissues and modulate disease behavior (Ferdoushi et al., 2021; Humphrey, 2017). Recent evidence indicates that neurons regulate tumor growth through neurotransmitter signaling and neurotrophin-mediated pathways, mirroring their roles in normal tissue homeostasis (Winkler et al., 2023). Conversely, tumor cells promote axonal outgrowth and neural remodeling via secretion of NGF, BDNF, and semaphorins (Silverman et al., 2021; Zhang et al., 2025a; Anderson and Simon, 2020). However, these cross-communications between tumor and neurons do not occur without the surveillance of the immune system which is the major and perhaps the first component of the TME (Wen et al., 2025) These interactions are not isolated but occur within a broader immunological context: immune cells such as macrophages and dendritic cells also contribute to neural remodeling via neurotrophin secretion (Huang et al., 2025; Yang et al., 2024; Renz et al., 2018a; Renz et al., 2018b). Importantly, the nature and consequences of neural-immune interactions appear tumor-type specific, and may vary based on autonomic input (sympathetic vs. parasympathetic) and disease stage. Current insights rely heavily on murine models, underscoring the need for human validation and functional studies using patient-derived tumors in humanized mouse models.

**FIGURE 2:**
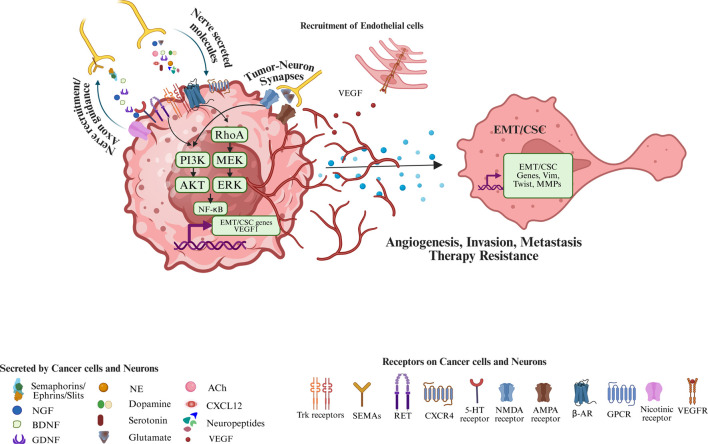
Neuron-tumor crosstalk as a driver of tumor progression and therapeutic resistance. Bidirectional interactions among tumor cells, peripheral/central neurons within the tumor microenvironment. Cancer cells and neurons release neurotrophins (NGF, BDNF, GDNF), chemokines (CXCL12), neurotransmitters (norepinephrine, acetylcholine, dopamine, serotonin, glutamate), neuropeptides, and axon-guidance molecules (semaphorins/ephrins/slits), which signal through cognate receptors on tumor and neural cells (Trk receptors, RET, CXCR4, β-adrenergic, nicotinic, GPCRs, 5-HT, NMDA/AMPA receptors) to activate downstream pathways (e.g., cAMP–PKA) that promote EMT/CSC plasticity, tumor cell survival, angiogenesis (VEGF–VEGFR), invasion, and metastasis. Created in BioRender. Korkaya, H. (2026) https://BioRender.com/r4qfx04.

The neural niche represents a specialized microdomain within the TME where tumor cells, infiltrating neurons, and immune elements engage in dynamic crosstalk (Figure 2). This niche is enriched in neurotrophins (e.g., NGF, GDNF), neurotransmitters (e.g., norepinephrine, acetylcholine), and axon-guidance molecules (e.g., netrins, semaphorins) that orchestrate cancer cell plasticity, angiogenesis, epithelial-mesenchymal transition (EMT), and stemness. Critically, neural signals directly modulate immune cell phenotypes: for example, adrenergic signaling promotes polarization of macrophages toward the M2/TAM phenotype (Park et al., 2024), enhances MDSC expansion (Mohammadpour et al., 2019), and induces T cell dysfunction (Qiao et al., 2021)by upregulating exhaustion markers (PD-1, TIM-3) and suppressing effector cytokine production (IFN-γ, granzyme B). Additionally, neurotransmitter receptors (e.g., β2-AR, α7nAChR, 5-HTRs) are differentially expressed on immune subsets, suggesting direct neural modulation of immunity. These signaling axes collectively foster an immunosuppressive and neuroinflammatory environment that protects tumor cells from immune clearance and limits immunotherapeutic efficacy.

Collectively, the neural–tumor–immune axis represents a unifying mechanism linking tumor progression, immune dysfunction, and treatment resistance. Understanding these interactions may reveal underexplored yet actionable therapeutic targets and enhance strategies to disrupt tumor-supportive neural circuits.

## Neural inputs and major ligand-receptor pairs

Neural input shapes both tumor-intrinsic behaviours and the immune landscape of the TME. Thorugh diverse neurotransmitters and neuropeptides, the nervous system communicates with tumor and immune cells in a receptor- and contect-dependent manner, regulating key processes such as antigen presentation, immune suppression, therapy resistance. Here, we provide a list of major neurotransmitters, neurotrophic factors and their receptors on tumors and immune cells involved in ligand-receptor-pathway interactions in the neural niche. Table 1 summarizes major neural ligands (neurotransmitters and neurotrophins), their receptors, activated downstream signaling pathways, cancer context, and immune/tumor effects.

**TABLE 1:** Neural ligands, receptors and downstream pathways in cancer progression.

Neural ligand	Key receptors (on tumor/Immune)	Major signaling pathways	Cancer context	Tumor/Immune outcomes
Norepinephrine (NE) *(Sympathetic neurotransmitter)*	β-Adrenergic receptors (β1, β2, β3) on tumor cells, endothelial cells, TAMs, MDSCs, T cellsetc.	cAMP/PKA activation; upregulation of VEGF, IL-6; activation of AKT, STAT3, NF-κB downstream	Breast, ovarian, pancreatic, prostate, melanoma; many solid tumors under stress	Immunosuppressive: Promotes M2 TAM polarization and MDSC recruitment; inhibits T/NK cell effector functions via PD-1 on T cells (Park et al., 2024; Liu et al., 2015; Sattler et al., 2025). Pro-tumor: Enhances angiogenesis and invasiveness, facilitating growth and metastasis as well resistance to anti-angiogenic therapy (Yang et al., 2009; Deng et al., 2014a)
Acetylcholine (ACh) *(Parasympathetic neurotransmitter)*	Muscarinic (M1-M5) acetylcholine receptors on tumor cells; Nicotinic ACh receptors on immune cells (e.g., macrophages) and neurons	Activation of PI3K/AKT, MEK/ERK, NF-κB apathways. Nicotinic (α7 nAChR on macrophages): JAK2/STAT3 and anti-inflammatory pathways	Gastric, colorectal, pancreatic, prostate cancers; immune cells in inflammatory conditions	Context-dependent: Can promote tumor growth via ACh-induced NGF release in gastric tumors, driving EMT and accelerating invasion via muscarinic signaling (Hayakawa et al., 2017). Conversely, cholinergic signaling in some contexts restrains tumorigenesis, possibly by activating anti-inflammatory signaling via vagal input suppressing cancer stemness via M1 muscarinic receptors (Renz et al., 2018a; Parteck et al., 2017). In immune cells, ACh (via nicotinic α7) inhibits pro-inflammatory TNFα release from macrophages, potentially reducing anti-tumor Th1 responses (Keever et al., 2023)
Dopamine *(Sympathetic catecholamine and neurotransmitter)*	D1-D5 dopamine receptors on tumor cells and immune cells	D1-like receptors (D1, D5): Gs/cAMP/PKA; can activate ERK. D2-like (D2-D4): Gi-mediated inhibition of cAMP; modulate PI3K/AKT and immune signaling	Glioblastoma, melanoma, ovarian, others; expressed on T cells and myeloid cells in TME.	Immune-modulatory (context-dependent): Dopamine can enhance anti-tumor immunity under some conditions such as D1 receptor signaling in T cells increases IL-2 production and their cytotoxic activity (Chovar-Vera et al., 2022). High dopamine levels may inhibit tumor angiogenesis and growth via vascular normalization and reprogramming of M2 macrophages in certain models (Qin et al., 2015a; Hoeppner et al., 2015). Conversely, dual activities were also reported (Ahangari and Norioun, 2025), some dopaminergic signals might suppress immunity via D2 receptors on immune cells (Nolan et al., 2020). Elevated dopamine and dopamine receptor is also implicated in hepatocellular carcinoma tumor growth (Yan et al., 2020)
Serotonin (5-HT) *(Neurotransmitter)*	5-HT receptors on tumor cells; some immune cells such as T cells and dendritic cells also express 5-HT receptors	Binding G-protein coupled receptors can activate ERK/MAPK or cAMP pathways; modulates cAMP in immune cells and can influence Ca^2+^ signaling	Gastrointestinal cancers (e.g., colorectal); some hormone-related cancers; tumor-associated platelets	Emerging evidence, conflicting findings: Serotonin and its receptors function in a context dependent manner in tumor growth (Zeng et al., 2025). It can promote tumor cell proliferation and migration in certain GI cancers via 5-HT-receptors and may skew immune responses (Schneider et al., 2021; Karmakar and Lal, 2021; Peters et al., 2020). Epidemiological studies of antidepressants (SSRIs, which raise 5-HT levels) have noted correlations with altered cancer risks/outcomes, but causality is unclear (Lee et al., 2023)
Glutamate *(Excitatory neurotransmitter)*	Glutamate receptors on cancer and neural cells: NMDA, AMPA, ionotropic receptors; metabotropic glutamate receptors (mGluR) in some tumors	NMDA/AMPA: Ca^2+^ influx, activation of CAMK, PI3K/AKT, MAPK; mGluR (GPCR) signaling modulates cAMP/PLC depending on subtype	Brain tumors: Gliomas and brain metastases of breast cancer and melanoma were shown to utilize glutamatergic signaling	Tumor-promoting (CNS context): Cancer cells can release and respond to glutamate, forming pseudo-synapses with neurons (Ren et al., 2025). In glioblastoma, neuron-to-tumor synaptic glutamate drives tumor proliferation and invasion via NMDA receptor signaling (Muller-Langle et al., 2019). This neuron-cancer cell synapses confers growth advantage and therapy resistance. Immune impact: Not well-characterized; both pro-tumor and anti-tumor functions reported. Glutamate may directly blunt cell-killing effects of neutrophils in the TME (Xiong et al., 2022). Conversely, GluR-T cell receptor signaling is shown to potentiate CD8^+^ T cell activation and effector function (de Aquino et al., 2025)
Substance P (SP) *(Sensory neuropeptide and pain fiber neurotransmitter)*	Neurokinin-1 receptor (NK1R) on tumor cells, endothelial cells, and immune cells such as macrophages	NK1R (GPCR) Gq/PLC and MAPK/ERK, NF-κB pathways; promotes Ca^2+^ signaling and cytokine release	Pancreatic cancer, head and neck cancer, and others with PNI; SP abundant in inflammatory conditions	Pro-tumor/pro-inflammation: SP/NK1R signaling promotes tumor proliferation, migration, and angiogenesis, and drives perineural invasion in some cancers (Ebrahimi et al., 2020; Chen et al., 2016). It also activates macrophages, leading to a pro-inflammatory milieu that paradoxically supports tumor growth (Singh et al., 2021). Blockade of SP/NK1R has shown tumor-suppressive effects in preclinical models, indicating this axis is a potential therapeutic target (Covenas et al., 2023)
Neuropeptide Y (NPY) *(Sympathetic co-transmitter)*	NPY receptors (Y1, Y2, Y5 subtypes) on tumor cells, blood vessels, and immune cells	Gi-coupled signaling (inhibits cAMP); can activate MAPK and angiogenic pathways via Y1	Breast cancer, ovarian cancer, neuroblastoma; often upregulated in hypoxic or stress conditions	Tumor-promoting: NPY signaling can enhance cancer cell migration, invasion, and neoangiogenesis (Abualsaud et al., 2020). For example, breast tumors upregulate NPY and its receptors, correlating with more aggressive behavior (Pascetta et al., 2023). Immune effects are less defined, but NPY can indirectly modulate immune cell migration via regulating hypoxia and has been linked to immunosuppressive environments. NPY is known to suppress T cell activation in other contexts (Chen et al., 2020)
Nerve Growth Factor (NGF) *(Neurotrophin)*	TrkA receptor (high-affinity) on neurons and some tumor cells; p75NTR (low-affinity NGF receptor) on various cells	TrkA activates RAS/MAPK/ERK, PI3K/AKT and PLCγ; induces genes for survival and axon growth. p75NTR can modulate NF-κB and RhoA pathways	Prostate, pancreatic, breast, and other malignancies: elevated NGF in tumors correlates with dense innervation; also in perineural invasion	Pro-neural, pro-survival: NGF is a key attractant for neurite growth into tumors driving tumor innervation in multiple malignancies (Lin et al., 2020; Hua et al., 2016; Pundavela et al., 2015). It can also directly enhance tumor cell survival and chemoresistance; e.g., NGF/TrkA signaling triggers EMT and therapy resistance in head and neck cancer cells (Lin et al., 2016). Immune effects: NGF shapes the microenvironment indirectly by increasing innervation and neuroimmune interactions, it contributes to immunosuppressive niche formation as targeting this axis potentiate immunotherapy (Yin et al., 2024). NGF can also promote neuroendocrine differentiation of tumor cells (as seen in prostate cancer), linked to immune evasion and treatment resistance (Chen et al., 2021). Targeting the NGF–TrkA axis (e.g., with neutralizing antibodies) not only reduces nerve infiltration and cancer cell invasion but may also mitigate pain and restore anti-tumor immune functions in ongoing studies (Yin et al., 2024)
Brain-Derived Neurotrophic Factor (BDNF) *(Neurotrophin)*	TrkB receptor expressed on neurons and some tumor cells (e.g., lung, breast) and also on certain immune cells (TrkB in subsets of T or NK cells has been reported)	TrkB activates PI3K/AKT and MAPK/ERK cascades; promotes cell survival, motility via Rac1, and differentiation. Also activates PLCγ mediated PKC pathway	Breast cancer, lung cancer, melanoma, others: TrkB often upregulated in metastatic or chemoresistant tumors. Also critical in brain tumors and perineural niches	Pro-metastatic: BDNF/TrkB signaling is known to prevent anoikis (detachment-induced apoptosis) of cancer cells, facilitating metastasis (Li et al., 2020; Kang et al., 2020). It can enhance tumor cell migration and invasiveness and is linked to poor prognosis when overexpressed (Bao et al., 2013). Immune interactions: BDNF may support regulatory immune cells in the TME; while direct immunomodulatory roles are not well-characterized, high BDNF levels often associated with an immunosuppressive, nerve-rich microenvironment (Liu et al., 2024). Blocking BDNF/TrkB pathway has shown reduced metastasis in models, making it a therapeutic interest alongside NGF (Li et al., 2015)
Glial Cell Line-Derived Neurotrophic Factor (GDNF) *(Neurotrophic factor)*	GDNF family receptor α (GFRα1) co-receptor and RET tyrosine kinase on neurons; some tumor cells express RET.	GDNF binding GFRα1 activates RET activates MAPK/ERK and PI3K/AKT pathways; supports neurite outgrowth and cell survival	Pancreatic cancer, prostate cancer, and others, especially in contexts of PNI. RET is implicated in tumor cell migration along nerves	Pro-invasive: GDNF/RET signaling strongly promotes nerve invasion by tumor cells (PNI). For instance, RET activation by GDNF drives pancreatic cancer cell migration toward nerves (He et al., 2014). RET signaling in tumors can also induce EMT and therapy resistance (Pecar et al., 2023). Immune aspects: By facilitating nerve-tumor structural interactions, GDNF contributes to creating “neuroimmune hubs” that favor tumor persistence (Huang et al., 2021). RET inhibitors can reduce PNI and tumor dissemination, potentially improving immune surveillance by disrupting this niche

### Sympathetic NE/β-adrenergic receptors (β-AR)

Norepinephrine released from sympathetic nerve terminals binds to β-AR (especially β_2_) on tumor cells, activating cAMP/PKA and downstream pathways (AKT, NF-κB, STAT3) that drive tumor proliferation, angiogenesis, invasion, and metastasis (Wang et al., 2025). This adrenergic signaling strongly promotes tumorigenesis via upregulating VEGF and MMPs, facilitating angiogenesis and matrix remodeling, while also inhibiting apoptosis, thereby giving cancer cells a growth and survival advantage (Qi et al., 2022).

### Parasympathetic ACh-muscarinic receptors

Acetylcholine from parasympathetic neurons binds muscarinic acetylcholine receptors on tumor cells. This triggers pro-invasive and pro-proliferative signals (via PI3K/AKT, MEK/ERK, and NF-κB pathways), leading to increased production of matrix metalloproteinases and other factors that enhance tumor cell motility and invasiveness (Zhang et al., 2025b). In some cancers, cholinergic signaling can also influence epithelial-mesenchymal transition (EMT) and metastatic dissemination (Fu et al., 2024; Zhao et al., 2015). Notebly, the impact of parasympathetic signaling appears contecxt-dependent. While it promotes invasion in gastric and colon cancer, it may also exert protective or suppressive effects in other malignancies. In prostate cancer, parasympathetic signaling has been linked to promoting later stages of tumor dissemination (Wang et al., 2021). These divergent roles underscore the need for tumor-type-specific analyses of cholinergic pathways.

### Sensory neuropeptide SP-NK-1 receptors

Substance P released by sensory nerve fibers binds to neurokinin-1 receptors (NK-1R) on cancer cells. Activation of NK-1R by SP potently promotes tumor cell proliferation and migration, stimulates angiogenesis, enhances cancer cell metabolism, and inhibits apoptosis in number of solid tumors (Ebrahimi et al., 2020; Chen et al., 2016; Huang et al., 2018). This SP/NK-1R signaling axis contributes to aggressive tumor behavior; for example, in pancreatic cancer, substance P–NK-1R signaling drives perineural invasion of cancer cells into nerves, a process associated with pain and metastasis (Zhang et al., 2025b; Ye et al., 2023). Tumor cells often overexpress NK-1R to exploit these pro-tumor effects of SP, and blocking SP-NK1R has been shown to suppress tumor growth (Chen et al., 2016; Zhang et al., 2025b; Munoz et al., 2020; Munoz and Covenas, 2020).

### Neuropeptide Y-NPY receptors (Y1/Y2/Y5)

Sympathetic nerves co-release neuropeptide Y (NPY), which can act on its receptors in the tumor microenvironment. NPY signaling is strongly linked to cancer progression, influencing tumor growth, migration, invasion, and blood vessel formation (Pascetta et al., 2023; Zhang et al., 2025b). For instance, upregulation of NPY Y1 and Y5 receptors in breast tumors correlates with greater cancer cell proliferation and migration (Pascetta et al., 2023). NPY/Y receptor axis also promotes angiogenesis in breast cancer by inducing VEGF (Medeiros and Jackson, 2013), cell survival and motility via activation of p44/42-MAPK pathway and RhoA signaling respectively in neuroblastoma (Abualsaud et al., 2020; Czarnecka et al., 2015).

## Nerve-immune crosstalk driving immune suppression

**FIGURE 3:**
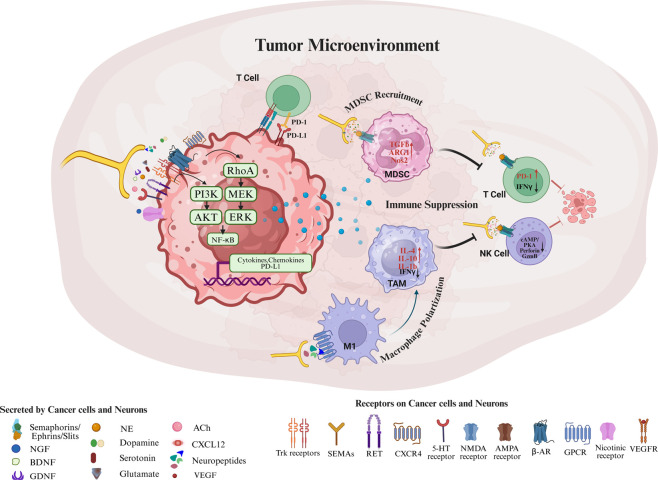
Neuronal regulation of tumor microenvironment. Cancer cells and neurons release neurotrophins (NGF, BDNF, GDNF), chemokines (CXCL12), neurotransmitters (norepinephrine, acetylcholine, dopamine, serotonin, glutamate), neuropeptides, and axon-guidance molecules (semaphorins/ephrins/slits), which signal through cognate receptors on tumor and neural cells (Trk receptors, RET, CXCR4, β-adrenergic, nicotinic, GPCRs, 5-HT, NMDA/AMPA receptors) to activate downstream pathways (e.g., cAMP–PKA) that promote tumor cell survival, angiogenesis, invasion and metastasis. These neural and tumor-derived cues reprogram the immune compartment by inducing M2/TAM polarization, expanding MDSCs, and suppressing cytotoxic T cell and NK cell effector functions (reduced IFN-γ, perforin, and granzyme B; increased IL-4, IL-10, IL-1β, TGF-β, ARG1, NOS2), thereby establishing an immunosuppressive niche that supports tumor progression and resistance to therapy. Created in BioRender. Korkaya, H. (2026) https://BioRender.com/r4qfx04.

The crosstalk between nerves, tumor cells, and immune cells can create an immunosuppressive niche that enables tumors to evade immune surveillance, promotes progression, and even confers resistance to therapies. Chronic stress and heightened sympathetic nerve activity (adrenergic signaling) are particularly implicated in driving these neuro-immune interactions (Inigo-Marco and Alonso, 2019). Key components of the immune system, including macrophages, myeloid derived suppressor cells (MDSCs), T lymphocytes, and natural killer (NK) cells, are modulated by neurotransmitters in ways that dampen anti-tumor immunity (Figure 3).

Sympathetic nerves release norepinephrine, which not only acts on tumor cells but also on immune cells. Norepinephrine binding to β-adrenergic receptors on tumor cells induces PD-L1 expression, a “do not kill me” signal that inhibits cytotoxic T cells (Wang et al., 2025). Adrenergic signals also act on infiltrating myeloid cells: for example, NE can recruit and activate MDSCs via β_2_-AR-mediated stress, causing these immunosuppressive cells to expand in number and function (Mohammadpour et al., 2019). MDSCs and tumor associated macrophages (TAMs) secrete immunosuppressive factors (IL-1β, IL-4, IL-10, TGF-β, etc.) and can express PD-L1 themselves, collectively blunting T cell and NK cell activity (Inigo-Marco and Alonso, 2019). Sensory neuropeptides such as substance P from nerve fibers can polarize macrophages toward a tumor-promoting M2 phenotype (Wang et al., 2025). The resulting TAMs release anti-inflammatory cytokines and support tumor growth while suppressing anti-tumor immune responses. This neuro-modulated immunosuppressive microenvironment not only accelerates tumor progression but also undermines immunotherapies and other treatments, as the immune system cannot effectively attack the cancer (Mohammadpour et al., 2019).

While these signaling interactions are well-characterized in murine models, further validation in human immune systems and across tumor types is needed to determine therapeutic feasibility. These insights not only advance mechanistic understanding but also provide rationale for therapeutic interventions that target neural-immune circuits to enhance immunotherapy.

### Adrenergic β-AR signaling upregulates PD-L1 on tumors

Norepinephrine (and epinephrine) signaling through β-adrenergic receptors on tumor cells leads to increased expression of PD-L1 on the cancer cell surface and blockade of β_2_-adrenergic receptors reduces cancer growth and enhances response to anti-CTLA4 therapy (Wang et al., 2025; Fjaestad et al., 2022). Elevated PD-L1 allows tumor cells to engage PD-1 receptors on T cells, turning off T cell responses and thereby enabling the tumor to escape cytotoxic T cell attack. NE-driven β-AR activation has been shown to directly suppress T cell activity in this manner, contributing to immune evasion (Wang et al., 2025; Nissen et al., 2018).

### Neurotransmitter-induced macrophage polarization (TAMs)

Neural inputs also influence macrophage polarization in the tumor microenvironment. Tumor associated macrophages can be skewed toward M2 (immunosuppressive) phenotype by neural factors (Wang et al., 2025). For example, substance P released by sensory nerves binds neurokinin-1 receptors (NK-1R) on macrophages, inducing M2 polarization (Lim et al., 2017). These M2 TAMs secrete anti-inflammatory cytokines (e.g., TGF-b, IL-4 and IL-10) and growth factors, while inhibiting Th1-type immune responses, thereby suppressing T cell and NK cell anti-tumor activity. Similarly, stress-induced norepinephrine (NE) activates β-adrenergic receptors on macrophages, driving M2-like gene expression programs in breast cancer (Qin et al., 2015b). In addition, neural signals may modulate dendritic cell differentiation and antigen presentation capacity, further compounding immune evasion.

### Suppression of T cell and NK cell function

Chronic activation of the sympathetic nervous (SNS) system negatively impacts adaptive and innate immune effector cells. T lymphocytes exposed to elevated NE levels exhibit reduced cytokine production (e.g., IFN-g), impaired proliferation, and increased expression of exhaustion markers such as PD-1, TIM-3 and LAG-3. v (Mohammadpour et al., 2019; Nissen et al., 2018). β2-adrenergic receptor activation on CD8^+^ T cells suppresses their cytolytic function and hampers memory formation. In line with this notion, NK cells experience functional inhibition through NE-mediated suppression of perforin and granzyme B release, mediated by cAMP/PKA signaling (Zhang et al., 2025a; Sun et al., 2018). These immunosuppressive effects culminate in decreased immune infiltration and impaired tumor clearance.

### Immune evasion and therapy resistance

The neuron-modulated immunosuppressive tumor microenvironment contributes to resistance against wide range of anti-cancer therapies. Catecholamines released during chronic stress inhibit immune-mediated tumor destruction and foster a TME dominated by suppressive myeloid cells. Tumors in high catecholamine contexts exhibit poor response to immune checkpoint blockade (ICB), chemotherapy, and radiotherapy (Zhang et al., 2025a; Mohammadpour et al., 2019; Inigo-Marco and Alonso, 2019). For instance, β-adrenergic signaling enhances DNA damage repair in tumor cells and upregulates PD-L1 expression, diminishing T-cell–mediated cytotoxicity (Inigo-Marco and Alonso, 2019; Nissen et al., 2018; Farooq et al., 2023; Oh et al., 2021). Studies have demonstrated that β-blockade reverses these effects: β-blockers reduce MDSC infiltration, restore T and NK cell function, and sensitize tumors to ICB. In NSCLC patients, concurrent use of β-blockers with ICB has been associated with prolonged progression-free survival, suggesting translational relevance (Oh et al., 2021). By blocking the stress pathways, T cells and NK cells can regain function, leading to improved anti-tumor immunity.

This reciprocal relationship adds a new layer to the tumor microenvironment’s complexity, sometimes described as the “neural niche” of the tumor (Termini et al., 2014). Importantly, neural involvement is not a rare occurrence; nerves are present in many solid tumors, and their density often correlates with more advanced disease or worse patient outcomes (Wang et al., 2021; Wang et al., 2024b).

The concept of a “neural niche” captures this reciprocal relationship between cancer and nerves, whereby neuronal activity drives immune suppression and tumor plasticity. This niche integrates neurotrophic factors, neurotransmitters, and immune regulators into a coordinated network that sustains tumor progression. Clinical evidence supports the oncogenic role of neural innervation: elevated intratumoral nerve density correlates with worse prognosis in cancers such as colon, rectal, breast, and head and neck carcinomas (Silverman et al., 2021). Intriguingly, spinal cord injury patients, who lose autonomic innervation to specific organs, show a markedly reduced risk of developing prostate cancer (Rutledge et al., 2017), emphasizing the dependence of some tumors on neural support.

### Neural contributions to tumor progression

The nervous system’s role in cancer progression spans from tumor initiation to metastasis. Neuronal signals can create a microenvironment conducive to transformation of cells with oncogenic mutations (Tibensky and Mravec, 2021). For example, in pre-malignant lesions of the pancreas and prostate, increased nerve fiber density has been observed, suggesting that neurogenesis and axonogenesis may be early events that support tumor development (Wang et al., 2021; Li et al., 2022). Growing tumors are often become richly innervated (Baraldi et al., 2022). Research has demonstrated that nerves actively infiltrate tumors in multiple cancer types (pancreatic, colorectal, prostate, breast, head and neck, among others) and that this innervation fuels tumor growth and metastasis (Wang et al., 2021; Zhang et al., 2025a; Li et al., 2022). Tumor-associated nerves with an increased neural activity facilitate tumor growth and metastasis by releasing growth-promoting molecules.

The bidirectional cancer-nerve communication allows them exchange signals which drives an increased neurogenesis of nerves while inducing aggressive properties of the tumor cells (Zhang et al., 2025a; Wen et al., 2025; Wu et al., 2023). Tumor cells *“hijack”* the nervous system by releasing neurotransmitters, neurotrophic factors, and axon guidance molecules that stimulate nerve growth or reprogram nearby neurons (Wang et al., 2021; Zhang et al., 2025a; Yang et al., 2024; Wang et al., 2024b; Li et al., 2022). One striking example is in prostate cancer: tumors can induce a switch in adjacent neurons, causing typically sensory nerves to acquire adrenergic (sympathetic) features that promote tumor progression (Wang et al., 2021; Ferdoushi et al., 2021; Cervantes-Villagrana et al., 2020). This phenomenon of neuronal reprogramming illustrates how cancer can manipulate nerve biology to its advantage, effectively creating a positive feedback loop. In line with this notion, head and neck cancers with loss of p53 was able to reprogram infiltrating neurons (Amit et al., 2020). Another example is in breast cancer brain metastases, where cancer cells have been found to co-opt neuronal synapses, they engage with glutamatergic synaptic inputs from brain neurons to receive growth signals via NMDA-type glutamate receptors (Neman et al., 2014). This direct synaptic interaction helps seeded cancer cells generate metastasis in the brain microenvironment.

Clinical and experimental denervation studies further validate the functional significance of nerves in cancer (Li et al., 2022; Espana-Ferrufino et al., 2018; Lei et al., 2017; Jiang et al., 2014; Deng et al., 2014b; Bapat et al., 2011). Surgical or chemical ablation of nerves in murine models reduces tumor growth and metastasis (Boilly et al., 2017). Patients with diminished autonomic tone such as vagotomy or nerve injury exhibit reduced cancer risk or improved survival in some context (Patel et al., 2011). Conversely, higher intra-tumoral nerve density in colon and rectal cancer patients has been associated with advanced stage and poorer survival (Albo et al., 2011). Together, these findings support the idea that many cancers are, to some degree, nerve-dependent, they rely on neural support or stimulation, a concept sometimes termed the “neural addiction” of cancer (Magnon and Hondermarck, 2023).

Neural regulation also intersects with cancer stemness and immune modulation. In triple-negative breast cancer, stemness-associated non-coding RNAs (lncRNA) correlate with distinct immune landscape and ICB responsiveness (Wu et al., 2023). These data imply that neurotrophin- and neurotransmitter-driven signaling in the TME may intersect with lncRNA-mediated stemness circuits to promote immune evasion, therapy resistance and clinical outcome (Feng et al., 2021). Integrating neurotrophic signaling, stemness, and immune biomarkers could therefore refine risk stratification and guide personalized therapeutic strategies.

### Systemic neuroendocrine networks in cancer

Emerging evidence supports the role of neuroendocrine stress responses as a critical systemic regulator of cancer progression, bridging central nervus system stress circuits with peripheral immune modulation. The hypothalamic-pituitary-adrenal (HPA) and sympathetic-adrenal-medullary (SAM) axes orchestrate this crosstalk, functioning as systemic extension of the tumor neural niche. Under chronic psychosocial stress or tumor-induced stress, elevated levels of cortisol and catecholamines reshape the tumor microenvironment and immune landscape tipping the balance toward tumor tolerance and therapy resistance.

This integration expands the understanding of tumor-nerve-immune crosstalk beyond local innervation to include brain-periphery signaling loops. Notably, the effects of chronic stress and systemic catecholamine/glucocorticoid signaling appear to be context-dependent, with tumor-type, disease stage and local immune landscape shaping the magnitude and directionality of the neuroendocrine influence. The hypothalamic-pituitary-adrenal (HPA) axis and the sympathetic-adrenal-medullary (SAM) axis, the body’s principal neuroendocrine stress systems, play key roles in modulating tumor biology (Vignjevic Petrinovic et al., 2023). Chronic activation of these axes (as seen in prolonged psychosocial stress or tumor-induced stress) leads to elevated glucocorticoids (cortisol) and catecholamines (epinephrine/norepinephrine), which can reshape the tumor microenvironment and immune surveillance (Khan et al., 2025). For example, persistent HPA/SAM activation can suppress antitumor immunity and promote a tumor-favorable milieu: stress-elevated catecholamines reduce the infiltration and efficacy of cytotoxic immune cells, inhibit cancer cell apoptosis, and induce epithelial-mesenchymal transition (EMT) and angiogenesis, collectively accelerating tumor growth, invasion and metastasis (Slominski et al., 2023). Glucocorticoids released from adrenal and extra-adrenal sources via HPA activation likewise exert immunosuppressive effects, dampening anti-tumor immune responses and skewing the balance toward tumor tolerance (Swatler et al., 2023; Slominski et al., 2024; Slominski et al., 2020). Importantly, these neuroendocrine stress signals have been linked to therapy resistance as tumors under chronic stress often exhibit poorer responses to chemotherapy and immunotherapy (Zhang et al., 2022a). Adrenergic signaling can upregulate survival pathways in cancer cells and impede immune-mediated tumor clearance, reducing treatment efficacy (Nissen et al., 2018; Zhang et al., 2024). Systemically, β-adrenergic signaling has been shown to suppress dendritic cell maturation, reduce antigen-presentation capacity and enhance regulatory (Treg) expansion, all contributing to a tolerogenic immune landscape that protects tumor from immune attack (Mohammadpour et al., 2019). Conversely, emerging studies suggest that blocking stress pathways can improve therapeutic outcomes: pharmacological inhibition of β-adrenergic receptors (β-blockers) or other adrenergic modulators can restore anti-tumor immune activity and has shown potential to enhance the efficacy of chemotherapy, immunotherapy, and targeted therapies (Fjaestad et al., 2022; Slominski et al., 2025). These findings underscore the clinical significance of neuroendocrine stress circuits and managing patient stress or pharmacologically modulating HPA/SAM activity may limit tumor progression and overcome therapy resistance.

While most mechanistic insights are driven from murine models, emerging retrospective clinical studies are beginning to validate the translational potential of neuroendocrine modulation in improving immunotherapy responsiveness and overall survival, particularly in cancers such as melanoma, NSCLC and breast cancer.

### HPA and SAM axes in cancer progression and immune modulation

HPA and SAM axis form a coordinated neuroendocrine interface between the brain, immune system, and peripheral tissues that tumors can exploit. Activation of the SAM axis (sympathetic nervous system and adrenal medulla) during stress leads to surges of epinephrine and norepinephrine, which bind β-adrenergic receptors on both tumor cells and various immune cells. This adrenergic stimulation has pleiotropic pro-tumor effects: it can directly stimulate cancer cell proliferation, survival, migration, and angiogenesis, and indirectly suppress anti-tumor immune functions (Zhang et al., 2024). For instance, β-adrenergic signaling skews immune cell differentiation and cytokine secretion toward an immunosuppressive profile, impairs the cytotoxicity of natural killer (NK) cells and T lymphocytes, and promotes the accumulation of pro-tumoral macrophages (Mohammadpour et al., 2019; Fjaestad et al., 2022). At the same time, stress-induced HPA activation elevates systemic cortisol, which further inhibits immune surveillance by inducing T cell apoptosis and impairing antigen presentation (Chen et al., 2022). Through these mechanisms, heightened activity of the HPA/SAM axes under chronic stress conditions creates an “immunosuppressive shield” around the tumor, allowing cancer cells to evade immune destruction and facilitating malignant progression (Chen et al., 2022; Zheng et al., 2025). In line with this, clinical and preclinical studies have observed that sustained psychosocial stress or depression (which often involves HPA axis dysregulation and hypercortisolism) is associated with faster tumor growth and metastasis in various cancers (Huang et al., 2025). Conversely, parasympathetic/vagal signaling, which often counterbalances sympathetic activity, may exert tumor-inhibitory effects in some contexts. For example, subdiaphragmatic vagotomy led to increased tumor growth and reduced survival in syngeneic models of murine pancreatic cancer (Renz et al., 2018a; Parteck et al., 2017). These observations highlight that the balance of autonomic tone influences cancer outcomes: sympathetic stress pathways generally promote tumor progression, whereas restoring parasympathetic activity or reducing adrenergic drive may improve anti-tumor immunity.

Stress-mediated immunosuppression and pro-survival signaling in tumors contribute to resistance against therapies. For instance, elevated catecholamine levels have been linked to reduced efficacy of immune checkpoint blockade and chemotherapy, partly by impairing T cell function and enhancing DNA damage repair in tumor cells (Zhang et al., 2024). On the other hand, disrupting these neuroendocrine signals can render tumors more vulnerable to treatment. Preclinical research and epidemiological data suggest that patients on β-blockers (adrenergic antagonists) sometimes show improved outcomes in certain cancers, presumably by abrogating adrenergic support of tumor growth and metastasis (Sharma et al., 2025). As noted above, pharmacological modulation of stress circuits, such as using β-adrenergic or α-adrenergic receptor blockers, is being explored to enhance the efficacy of chemotherapy, immunotherapy, and targeted therapy in cancer patients (Carnet Le Provost et al., 2023; Kokolus et al., 2018). Additionally, behavioral or psychosocial interventions that reduce chronic stress (exercise, meditation, counseling) could theoretically dampen HPA/SAM overactivity and thereby bolster the patient’s endogenous anti-tumor immunity. Together, these insights into the HPA and SAM axes underscore a paradigm in which cancer is not only a disease of genetic mutations but also a systemic maladaptation of stress-immune homeostasis.

### Induction and mobilization of immunosuppressive MDSCs by systemic neuroendocrine network

Catecholamines released by sympathetic nerves can bind β_2_-adrenergic receptors on myeloid cells, notably on myeloid derived suppressor cells (MDSCs) (Mohammadpour et al., 2019; Mohammadpour et al., 2021). Chronic adrenergic stimulation significantly increases MDSC recruitment, survival, and immunosuppressive function in tumors (Mohammadpour et al., 2019; Farooq et al., 2023). Under β-AR signaling, MDSCs upregulate enzymes like arginase-I and inhibitory ligands like PD-L1, and more effectively suppress T cell proliferation (Mohammadpour et al., 2019; Schuster et al., 2023). β-Adrenergic agonists were also shown to activate inflammatory signaling via TLR2 which may play role in MDSC induction and expansion (Agac et al., 2018; Manni et al., 2011). These stress-expanded MDSCs produce IL-10, TGF-β and other factors that inhibit effector lymphocytes and even impair NK cell function (Mohammadpour et al., 2019; Inigo-Marco and Alonso, 2019). Collectively, expansion of immune suppressive myeloid cells and diminished cytotoxic lymphocytes will lead to an immunosuppressive microenvironment dominated by MDSCs and M2-polarized macrophages.

## Tumor innervation and perineural invasion

**FIGURE 4:**
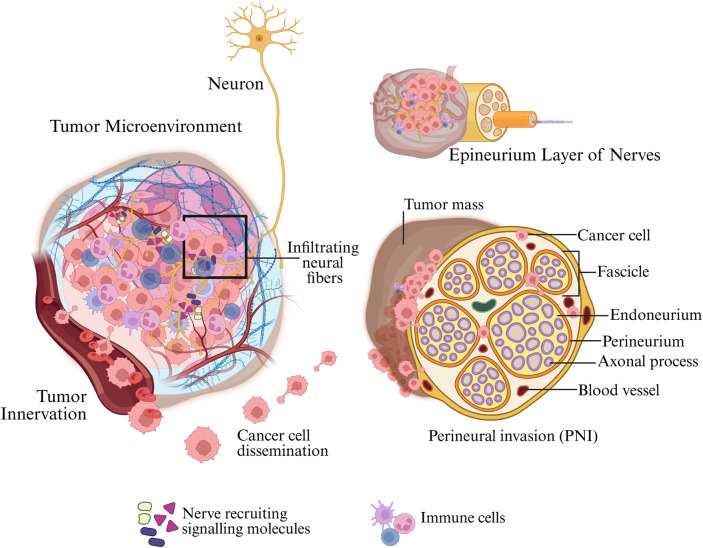
Tumor–nerve interactions leading to neural infiltration and perineural invasion. The tumor microenvironment secretes nerve-recruiting signalling molecules (e.g., NGF, BDNF, GDNF), which attract nearby neurons and promote the extension of infiltrating neural fibers into the tumor mass. Immune cells, stromal elements, and vascular structures further support this neurotrophic niche. The boxed region highlights the local accumulation of neurotrophic factors and early axonal ingrowth toward cancer cells. As neural fibers penetrate the tumor, cancer cells disseminate along these neuronal tracks and migrate toward peripheral nerves. On the right, a cross-sectional view of a peripheral nerve illustrates cancer cell invasion into the epineurium, perineurium, and endoneurium layers, representing the process of perineural invasion. Created in BioRender. Korkaya, H. (2026) https://BioRender.com/r4qfx04.

Cancer-nervous system interaction primarily manifests by two forms: perineural invasion (PNI) and tumor innervation. Both represent physical manifestations of cancer–neuron crosstalk in the tumor microenvironment (Figure 4).

### Perineural Invasion (PNI)

PNI occurs when cancer cells actively invade the spaces surrounding nerves, often migrating along the nerve sheath. Histopathologically, PNI is typically defined by the presence of tumor cells within the perineural space or encircling the nerve sheath (Chavan et al., 2017). PNI can be further subclassified into purely perineural (tumor outside but circumferentially surrounding the nerve) and intraneural (tumor breaching the perineurium and infiltrating within the fascicles), with the latter often reflecting more advanced disease (Chen et al., 2019; Wang et al., 2024b; Liang et al., 2016). Grading schemes commonly distinguish: Grade 0 (no PNI), Grade 1 (perineural involvement without intraneural spread), and Grade 2 (overt intraneural invasion with distortion of nerve architecture).

PNI is a common feature in many malignancies, notably pancreatic, head and neck, prostate, and colorectal cancers, and is strongly associated with metastasis, recurrence, and pain (Huang et al., 2025). Tumor cells that disseminate via nerves can travel to distant sites by tracking along nerve bundles which is distinct from circulation in lymphatics or bloodstream, and it often correlates with especially aggressive disease. Clinically, the detection of PNI in a tumor biopsy is considered an adverse prognostic indicator for number of solid tumors (Nozzoli et al., 2024; Narayan et al., 2021). For example, in pancreatic ductal adenocarcinoma, PNI is extremely prevalent and contributes to severe pain experienced by patients, as well as it is associated with earlier local recurrence after surgery (Liang et al., 2016).

Cancer cells in PNI exhibit invasive behavior which is driven by signals from the nerves and Schwann cells that foster a permissive niche. Indeed, recent work shows that PNI formation is orchestrated by an array of molecular and cellular interactions: neurotrophins, chemokines, and cytokines provide chemical cues, while Schwann cells and immune cells (like macrophages and T cells) actively participate in enabling cancer cells to infiltrate nerves (de Visser and Joyce, 2023; Zhang et al., 2025a). This multifactorial coordination not only facilitates tumor spread along nerves but also leads to neuropathic cancer pain, as nerve integrity is compromised.

### Tumor innervation (Neoneurogenesis)

Tumor innervation refers to the growth of new nerves into the tumor tissue via the tumor cell secreted factors which attract neuronal axons inducing nerve sprouting within the tumor (Payton, 2013). This result in increased nerve fiber density within the tumor. This kind of neo-innervation has been reported in number of solid tumors including prostate cancer, breast cancer and gastric cancer (Wang et al., 2021; Wu et al., 2023; Nozzoli et al., 2024). For instance, prostate tumors in mouse models have been shown to stimulate dense sympathetic and parasympathetic nerve ingrowth, which in turn enhances tumor progression and dissemination (Silverman et al., 2021; Magnon et al., 2013). Mechanistically, tumor-produced growth factors serve as chemoattractants for neurites. Histological examination of tumors often reveal nerve fascicles penetrating into the tumor stroma; these nerves may form synapse-like structures with cancer cells or release neurotransmitters locally (Nguyen et al., 2023). Tumor innervation often accompanies PNI in a vicious cycle, as tumor cells invade nerves, they can also prompt further nerve growth, generating a tumor-nerve network. Both PNI and active innervation are indicators of a high degree of tumor-nerve interplay, and both are linked to more aggressive tumor phenotypes (Gysler and Drapkin, 2021). Advent of advanced spatial imaging and tracing technologies facilitated the deeper characterization of these tumor-nerve networks demonstrating that some tumors establish long-distance connections with the nervous system. For example, nerves from ganglia or the spinal cord may extend processes into a tumor, potentially allowing central neural influences to directly impinge on tumor cells (Wang et al., 2021; Reavis et al., 2020).

Extensive studies collectively suggest that PNI and tumor innervation are two sides of the cancer-neuron interaction. PNI reflects tumor cells moving into neural territory, whereas innervation reflects nerves growing into the tumor territory. Both phenomena often coexist and reinforce one another. Clinically, their presence underscores the need to consider nerves as both diagnostic markers and potential therapeutic targets in cancer management.

Beyond local neural remodeling, neurogenic signals can influence systemic dissemination by modulating circulating tumor cells (CTCs). CTCs constitute a critical intermediate between primary tumors and distant metastases and are now monitored using a variety of *in vivo* and *in vitro* detection platforms that also inform immunotherapy response (Zhan et al., 2023). Stress-related adrenergic signaling and neurotrophin-rich microenvironments may enhance CTC survival, epithelial–mesenchymal plasticity and immune escape, thereby linking the neural niche to the systemic tumor–immune interface (Triaca et al., 2019). Although the studies are limited on this area, incorporating CTC metrics into cancer-neuron-immune crosstalk will be essential to fully understand and therapeutically target the metastatic cascade.

## Neurotrophic factors and axon guidance

Neurotrophic factors are growth factors that support the survival and growth of neurons. Cancers often exploit these molecules to attract nerve infiltration and to receive pro-survival signals from them. For example, tumor and stromal cells secrete nerve growth factor (NGF) and brain-derived neurotrophic factor (BDNF) – two well-known neurotrophins–which can stimulate nearby nerve fibers to sprout and innervate the tumor (Blondy et al., 2019). NGF and BDNF bind to Trk family receptors (TrkA and TrkB, respectively) on neurons, activating pathways that drive neurite extension. In pancreatic cancer and prostate cancer, elevated levels of NGF in the tumor microenvironment correlate with increased nerve density and PNI, implicating NGF/TrkA signaling in promoting tumor-associated neurogenesis Similarly, GDNF (glial cell line-derived neurotrophic factor) and its family member artemin are produced in certain tumors; they bind the RET receptor on nerves to induce robust axonal growth towards tumors (Sun et al., 2024). This GDNF–RET pathway has been shown to accelerate perineural invasion in pancreatic cancer models, and blocking GDNF release from nerves or inhibiting RET signaling can prevent PNI in experimental settings (Sun et al., 2024; Amit et al., 2019). Such findings suggest that targeting neurotrophic signaling (for example, with RET inhibitors or Trk antagonists) could disrupt the neurotropic attraction between tumors and nerves.

In addition to feeding nerve growth, neurotrophins can directly influence cancer cells. Many tumors express receptors for neurotrophic factors; for instance, TrkB (the receptor for BDNF) is found on various carcinoma cells and can drive tumor cell migration, survival, and even neuron-like properties in cancer cells when activated (Thiele et al., 2009). There is evidence that BDNF-TrkB signaling in tumor cells activates pro-survival pathways (MAPK/ERK, PI3K/Akt) and may contribute to metastasis and resistance to anoikis (Hua et al., 2016). In prostate cancer, NGF has been reported to promote neuroendocrine differentiation of tumor cells, a phenotype associated with therapy resistance, via interactions with muscarinic receptors (Chen et al., 2021). Thus, neurotrophic factor signaling creates a feed-forward loop: tumors secrete neurotrophins to attract and grow nerves, and in turn those neurotrophins (from either the tumor or the new nerves) can activate tumor cell pathways that enhance malignancy.

Axon guidance molecules, including netrins, semaphorins, ephrins, slits and chemokines, are aberrantly expressed in tumors and influence nerve and cancer cell positioning (Nourisanami et al., 2024; Nakamoto et al., 2004). Semaphorin 4F and Netrin-1 promote neural invasion in pancreatic and colon cancers by creating gradients that attract nerves or cancer cells (Sun et al., 2024; Akino et al., 2014). Chemokines like CXCL12 (SDF-1) secreted by nerves or stromal cells can draw cancer cells towards nerves, as shown in pancreatic cancer where dorsal root ganglia–derived CXCL12 attracted tumor cells to nerve vicinities (Sun et al., 2024; Xu et al., 2015). Adhesion molecules such as L1CAM normally involved in neuron pathfinding are co-opted by cancer cells to facilitate their migration along nerves; blocking L1CAM impairs PNI in pancreatic cancer models (Zhang et al., 2022b). By targeting these pathways such as neutralizing NGF or inhibiting CXCL12/CXCR4 axis, researchers aim to interrupt the communication between nerves and cancer cells (Figure 4).

## Neurotransmitters and adrenergic signaling

Neurotransmitters are crucial mediators of cancer-neuron crosstalk. Tumor tissues are often infiltrated by autonomic nerve fibers (sympathetic and parasympathetic) that release neurotransmitters such as norepinephrine, epinephrine, acetylcholine, dopamine, serotonin, glutamate, and other transmitters (Wang et al., 2021; Silverman et al., 2021; Tibensky and Mravec, 2021). These molecules can bind to their respective receptors on cancer cells or immune cells in the TME and modulate tumor aggressiveness.

A well-documented example is adrenergic signaling mediated by the sympathetic nervous system. Chronic stress or tumor-associated neurogenesis can lead to elevated local levels of norepinephrine (NE) and epinephrine, activating β-adrenergic receptors (β-ARs) on tumor cells and stromal cells (Wang et al., 2021; Zhang et al., 2024). β-adrenergic pathways in cancer promote tumor growth, angiogenesis, invasion, and immunosuppression (Zhang et al., 2024; Hong et al., 2021). In stress-induced breast and ovarian cancer models, norepinephrine drives metastasis to distant organs, effects mimicked by pharmacological β-AR agonists and blocked by β-AR antagonists (Zhang et al., 2024; Conceicao et al., 2021). Mechanistically, β-adrenergic signaling upregulates VEGF expression, stimulating angiogenesis via recruiting VEGFR + endothelial cells (Al Khashali et al., 2023). It also modulates the immune microenvironment; norepinephrine acting on β_2_-adrenergic receptors on immune cells inhibits anti-tumor immunity by reducing interferon-γ production in T cells and promoting an immunosuppressive phenotype (Wang et al., 2021; Tha et al., 2023). β-adrenergic activation increases infiltration of tumor-promoting macrophages (TAMs), impairs immune therapy responses and induces metastasis-associated gene signatures, whereas using β-blockers reverses these changes (Huang et al., 2025; Bruno et al., 2023). In pancreatic cancer, sympathetic nerves release norepinephrine that recruits macrophages via β-adrenergic signals; these macrophages facilitate tumor progression and therapy resistance (Wang et al., 2021; Sattler et al., 2025).

Parasympathetic (cholinergic) signaling can also influence tumors in a context-dependent manner. Parasympathetic nerves release acetylcholine (ACh) which acts on muscarinic or nicotinic receptors. In some cancers (e.g., gastric cancer), cholinergic stimulation via vagus nerve activity increases tumor growth: ACh can induce NGF production in gastric epithelium, accelerating tumor progression (Hayakawa et al., 2017). In other contexts, parasympathetic input may restrain tumor progression: in pancreatic cancer, vagal innervation appears to counteract the pro-tumor adrenergic effects, as surgical vagotomy led to faster tumor growth and worse survival in mouse models (Renz et al., 2018a). These observations suggest a balance between sympathetic and parasympathetic influences, sometimes termed the “yin-yang” of autonomic regulation in cancer (Wang et al., 2021). In prostate cancer, sympathetic nerves predominantly drive early tumor proliferation via β-adrenergic signaling, while parasympathetic nerves (via muscarinic receptors) contribute to later dissemination and metastasis (March et al., 2020). The net effect of autonomic neurotransmitters depends on receptor expression patterns and downstream pathways in tumor and stromal cells.

Other neurotransmitters that may also play roles in the tumor microenvironment.

### Dopamine

Dopamine receptors are expressed on tumor and immune cells in several solid tumors. Depending on receptor subtype and context, dopamine can inhibit angiogenesis and tumor growth via D2 receptor-mediated suppression of VEGF signaling or support tumor-promoting inflammation through D1-coupled pathways (Peters et al., 2020; Sobczuk et al., 2020). On immune cells, dopamine modulates T cell activation and NK cell cytotoxicity, indicating that dopaminergic signaling can drive anti-tumor immunity (Chovar-Vera et al., 2022). In contrast, elevated D2 dopamine receptor (DRD2) is shown to drive tumor progression via hypoxia-inducible factor-1α (HIF-1α) in mouse melanoma tumor model (Liu et al., 2021).

### Serotonin (5-HT)

Serotonin functions as a growth and motility factor in multiple epithelial cancers through 5-HT receptors and downstream ERK/AKT signaling (Karmakar and Lal, 2021; Peters et al., 2020). It also shapes the tumor immune microenvironment by regulating myeloid cell recruitment, T cell function and cytokine production; attenuation of peripheral serotonin inhibits tumor growth and improves responses to immune checkpoint blockade in preclinical models (Schneider et al., 2021; Karmakar and Lal, 2021).

### Glutamate

Glutamatergic neurotransmission is particularly relevant in primary brain tumors and brain metastases. Gliomas and metastatic breast cancer cells can release and respond to glutamate, engaging AMPA and NMDA receptors to drive proliferation, invasion and synapse-like contacts with neurons (Zeng et al., 2019; Sontheimer, 2008). These neuro-cancer synapses support electrical and metabolic coupling that enhances malignant growth and may confer resistance to therapy.

### Neuropeptides

Neuropeptides such as substance P, calcitonin gene-related peptide (CGRP), vasoactive intestinal peptide (VIP) and neuropeptide Y (NPY) are released by sensory and autonomic fibers and act on GPCRs expressed by tumor, endothelial and immune cells (Sun et al., 2024; Shalabi et al., 2024). Substance P–NK1 signaling promotes cancer cell migration, angiogenesis and mast cell activation; CGRP and VIP can suppress anti-tumor immune responses by inhibiting dendritic cell maturation and T cell activation. Targeting neuropeptide–receptor axes therefore represents another opportunity to modulate both tumor and immune compartments of the neural niche.

Tumors innervation may indeed release a cocktail of neurotransmitters into the TME, while tumor cells also produce or respond to these neurotransmitters. The outcome is a neurochemical dialogue that can significantly skew tumor biology and the clinical outcome for the patients. Blocking specific neurotransmitter receptors on tumor cells (for example, using β-adrenergic blockers, or muscarinic receptor antagonists) has emerged as a potential strategy to disrupt the pro-tumor effects of nerves. Moreover, stress reduction and β-blockade have been investigated clinically to see if they can improve cancer outcomes by dampening adrenergic signaling.

## Experimental models demonstrating neural control of tumor growth

A range of experimental systems have demonstrated that nerves are not only bystanders but active drivers of tumor initiation, progression, and dissemination.

### Surgical denervation

In prostate cancer models, surgical sympathectomy reduces early tumor incidence, while pelvic parasympathetic denervation diminishes distant metastasis, indicating stage-specific roles for autonomic inputs (Magnon et al., 2013). Vagotomy or local botulinum toxin–mediated cholinergic blockade suppresses gastric tumorigenesis and prolongs survival, supporting a critical role of cholinergic signaling in gastric cancer progression (Rabben et al., 2021; Zhao et al., 2014). In pancreatic cancer, experimental vagotomy accelerates tumor growth whereas sympathetic ablation reduces cancer stemness and tumor burden, highlighting the importance of autonomic balance rather than unidirectional effects (Renz et al., 2018a; Parteck et al., 2017; Saloman et al., 2016).

### Chemical denervation

Chemical sympathectomy with 6-hydroxydopamine (6-OHDA) depletes norepinephrine-containing fibers and reduces stress-induced tumor growth and metastasis in breast and ovarian cancer models (Zhang et al., 2024; Conceicao et al., 2021; Szpun et al., 2016). TRPV1-targeted approaches or capsaicin-based sensory denervation blunt neurogenic inflammation and attenuate nerve-facilitated invasion in pancreatic and colorectal cancer (Zhang et al., 2025c; Schwartz et al., 2011).

### Genetic models

Genetic disruption of neural growth or signaling further supports a causal role of nerves. Deletion or pharmacological inhibition of RET or Trk family receptors (TrkA/B) impairs GDNF- and NGF-driven axonogenesis toward tumors and reduces perineural invasion in pancreatic and head and neck cancer models (Lin et al., 2020; Hua et al., 2016; Thiele et al., 2009). Tumor or host-specific β_2_-adrenergic receptor (Adrb2) deletion protects against chronic stress-enhanced tumor growth and reverses β-adrenergic gene signatures associated with metastasis (Renz et al., 2018b; Al Khashali et al., 2023). Knockdown of NGF or its receptors in gastric and prostate cancer models decreases tumor nerve density, neuroendocrine differentiation, and PNI, further illustrating a feed-forward neurotrophic loop (40,61,73). Furthermore, adrenergic nerve-derived noradrenaline in the prostate cancer stroma was shown to activate an angiogenic switch fueling tumor growth via alteration of endothelial cell metabolism (Zahalka et al., 2017). Deletion of endothelial Adrb2 gene inhibited angiogenesis in the tumor stroma and reduced prostate cancer progression.

### β-adrenergic blockade

A growing body of evidence demonstrates that β-adrenergic signaling regulates both tumor cell and the microenvironment, integrating neural inputs into cancer progression. Across multiple cancer types, chronic activation of β-adrenergic receptors (β-ARs) not only enhances tumor cell intrinsic programs but also profoundly reshapes antitumor immunity. Mechanistically, β-AR stimulation directly impairs cytotoxic CD8^+^ T cell expansion, IFNγ production, and killing capacity, thereby diminishing responsiveness to immunotherapies (Zhang et al., 2024; Carnet Le Provost et al., 2023). At the tumor cell level, β-adrenergic agonists increase traction forces, stiffness, and invasive potential in triple-negative breast cancer cells through a βAR–RhoA–ROCK–myosin II axis, demonstrating that stress-related catecholamines mechanically prime cancer cells for metastasis (Kim et al., 2025). Conversely, β-blockade with propranolol reveals the extent to which this neural signaling suppresses antitumor immunity: inhibiting β-ARs drives a potent Th1-polarized cytotoxic CD4^+^ T cell response, reduces monocyte-mediated immunosuppression, and significantly attenuates metastasis in multiple tumor models while synergizing with CTLA-4 blockade (Fjaestad et al., 2025). Pharmacological blockade of β-adrenergic receptors with non-selective (propranolol) or selective (atenolol, metoprolol) β-blockers mitigates tumor growth, angiogenesis, invasion, and recruitment of tumor-promoting macrophages across several models (Fjaestad et al., 2022; Carnet Le Provost et al., 2023). Combining β-blockers with immune checkpoint inhibitors enhances anti-tumor T cell function and reduces exhaustion markers (Fjaestad et al., 2022; Globig et al., 2023), indicating that neural targeting can reprogram the immune landscape. Together, these studies position β-adrenergic signaling as a central node linking neural activity to tumor progression, mechanical adaptation, and immune evasion, implicating adrenergic blockade as a promising therapeutic strategy to disrupt neuro-immune circuits.

Collectively, these denervation, β-blockade, and gene-knockout models demonstrate that autonomic and sensory nerves are functionally required for full malignant progression in many solid tumors, supporting the concept of “neural addiction” in cancer (Boilly et al., 2017; Magnon and Hondermarck, 2023).

## Emerging therapeutic insights

Improved understanding of cancer-neuron interactions has accelerated efforts to therapeutically target this crosstalk. Strategies include β-adrenergic blockade, inhibition of neurotrophic pathways, denervation and neuromodulation.

Perhaps the most advanced approach is repurposing β-blocker drugs (commonly used for cardiac conditions) for cancer therapy. Preclinical studies show that propranolol and other β-adrenergic antagonists can slow tumor growth and metastasis in models of breast, ovarian, and pancreatic cancer by blocking stress-mediated tumor activation (Carnet Le Provost et al., 2023). In preclinical mouse models, β-blockers reduced infiltration of M2-like macrophages and dampened the expression of pro-metastatic genes in the primary tumor (Fjaestad et al., 2022). There is also evidence that β-blockade can enhance the efficacy of other treatments: for example, using a β_1_-selective blockers (e.g., atenolol) in combination with anti-PD-1/PD-L1 immunotherapy improved T cell function and reduced immune exhaustion in mice, thereby augmenting the anti-tumor immune response (Fjaestad et al., 2022; Globig et al., 2023). Retrospective analyses have noted that cancer patients who happen to be on β-blockers for other indications sometimes have better outcomes (Nagaraja et al., 2013). One study found that patients with high-risk or metastatic prostate cancer on β-blockers showed prolonged survival compared to those not on β-blockers (Zahalka et al., 2020). Similar associations have been reported in breast cancer cohorts (Powe et al., 2010). These findings have led to multiple clinical trials investigating perioperative or adjuvant propranolol in cancers such as melanoma, breast cancer, and angiosarcoma. While results are still emerging, beta-adrenergic inhibition is a promising strategy to counteract tumor-promoting stress signals.

Targeting Neurotrophic Pathways offers another approach. Experimental therapies include inhibitors of the RET kinase (to block GDNF/RET signaling) and Trk inhibitors (to block NGF/TrkA or BDNF/TrkB signaling) (Drilon et al., 2017). In pancreatic cancer models, RET inhibition reduced PNI and tumor spread along nerves (Sun et al., 2024; Gasparini et al., 2019). Neutralizing antibodies against NGF, originally developed for pain management, may simultaneously reduce nerve infiltration and cancer-associated pain if applied in oncology (Jaffal and Khalil, 2024). Silencing NGF or blocking its receptor impedes the attraction of nerves to tumors (Hayakawa et al., 2017; Chen et al., 2021; Sun et al., 2024). Targeting the GDNF–RET axis is supported by findings that nerve-derived GDNF is key for PNI; thus, RET inhibitors (already in use for RET-mutated cancers) could be repurposed to prevent nerve invasion in cancers with PNI (Amit et al., 2019; Lin et al., 2017).

Denervation Strategies directly target neural input to tumors. Preclinical studies have shown that surgical cutting nerves or blocking neural input to an organ can retard tumorigenesis (Saloman et al., 2016). In line with the notion, surgical vagotomy (severing the vagus nerve) or local injection of botulinum toxin (to silence cholinergic signaling) significantly suppressed the growth of gastric tumors and improved survival in mouse models (Zhao et al., 2014). Similarly, in prostate cancer models, chemical sympathectomy (destroying sympathetic nerves) reduced early tumor incidence, and cutting pelvic parasympathetic nerves reduced metastasis to distant sites (Magnon et al., 2013). While surgical denervation is not a routine cancer therapy, these findings suggest that regional nerve blockade could be beneficial. Some clinicians have considered or piloted celiac plexus neurolysis (a procedure that destroys nerves to treat pancreatic cancer pain) to see if it might also impact tumor progression (Arcidiacono et al., 2011). However, the challenge is achieving tumor-specific denervation without unacceptable side effects; hence, targeted chemical denervation (using drugs or gene therapy to ablate nerves in the tumor vicinity) is an area of active research.

Beyond β-blockers, other drugs that affect the nervous system are being evaluated for anticancer properties. For example, α2-adrenergic receptor agonists and antagonists (which modulate norepinephrine release) might influence tumor vascular dynamics. Dopamine agonists have anti-angiogenic effects that could be harnessed in certain cancers. Anti-depressants that alter serotonin levels have been observed in some epidemiological studies to correlate with cancer risk or progression, although causality is unclear (Lee et al., 2023). Moreover, drugs targeting sensory neurons (like TRPV1 inhibitors, which might reduce neurogenic inflammation) could potentially reduce nerve-driven tumor promotion and pain simultaneously. The concept of “neuroimmune modulation” describing how nerves influence immune cells in the TME is also gaining traction. For instance, activating certain neural pathways can alter macrophage polarization or T cell activity in tumors (Zhang et al., 2025a; Wen et al., 2025). There is interest in exploiting this by either blocking pro-tumor neural signals or activating anti-tumor neural reflexes.

To provide a practical overview of neural-targeted strategies, we summarize the main targets, experimental models, efficacies observed and safety considerations in Table 2.

**TABLE 2:** Neural-targeted therapeutic strategies in cancer.

Agent class	Molecular target and mechanism	Representative models/Tumor types	Major anti-tumor effects	Key safety/Translational considerations
β-blockers (propranolol, atenolol, metoprolol, etc.)	Block β-adrenergic receptors; reduce stress-mediated catecholamine signaling in tumor and immune cells	Breast, ovarian, pancreatic, prostate, melanoma models; retrospective clinical cohorts	↓Tumor growth, angiogenesis and metastasis ↓TAM/MDSC recruitment; improved T cell function; synergy with anti-CTLA-4 and anti-PD-1/PD-L1	Generally well-tolerated; cardiovascular monitoring; optimal timing and combinations with immunotherapy under investigation
RET inhibitors (e.g., selpercatinib, pralsetinib)	Inhibit GDNF–RET signaling driving axonogenesis and PNI	Pancreatic, head and neck, thyroid cancer models; RET-altered solid tumors	↓ PNI and neural outgrowth; direct anti-tumor effects in RET-mutant cancers	On-target toxicities similar to other TKIs; need for patient selection (RET expression/mutation); potential adjunct in PNI-high tumors
Trk inhibitors (e.g., entrectinib, larotrectinib; next-generation TRK inhibitors)	Block NGF–TrkA and BDNF–TrkB signaling in neurons and, where expressed, tumor cells	NTRK fusion–positive tumors; preclinical PNI and innervation models	Direct anti-tumor activity in fusion-positive tumors; potential to reduce neoneurogenesis and stemness	Resistance mutations and CNS penetration issues; potential repurposing in Trk-expressing, non-fusion tumors
Anti-NGF antibodies/NGF pathway blockade	Neutralize NGF or inhibit NGF–TrkA signaling to reduce nerve growth and tumor–nerve coupling	Pancreatic, gastric, prostate models; pain models	↓ Tumor nerve density, PNI and cancer-associated pain; reduced neuroendocrine differentiation	Risk of sensory neuropathy and altered nociception; careful dosing and patient selection needed
Surgical/chemical denervation (vagotomy, sympathectomy, botulinum toxin, 6-OHDA)	Interrupt autonomic or sensory innervation to target organ	Gastric, pancreatic, prostate models	Suppression of tumor initiation and/or progression; reduced PNI and local invasion	Invasive or irreversible in many cases; organ-specific side effects; may best suit localized, high-risk settings or be combined with systemic therapies

These therapeutic insights are still being tested, but collectively they herald a new “neuro-targeted” paradigm for cancer treatment. Just as the rise of immunotherapy revolutionized cancer care by targeting the immune microenvironment, targeting the neural microenvironment could become another pillar of therapy. It is notable that combining neural-targeted approaches with existing treatments might yield the best results, for example, stress-reducing interventions and β-blockers could enhance immune checkpoint inhibitors by preventing stress-induced immunosuppression. Ongoing clinical trials and future studies will clarify which neural interventions are most feasible and effective in patients.

## Controversies, unresolved questions and future directions

Despite significant advances in characterizing tumor-neuron-immune interactions, many fundamental questions remain unanswered. Here we outline key controversies, gaps in knowledge, limitations of current models, and conflicting findings.

### Complexity of neural inputs

Tumors are innervated by multiple nerve types (sympathetic, parasympathetic, sensory), but their relative contributions can vary by cancer type and stage. For instance, sympathetic adrenergic signaling often promotes early tumor growth and angiogenesis, whereas parasympathetic (cholinergic) signals have been reported to either facilitate late-stage metastasis or, conversely, to inhibit tumor progression in certain contexts (Renz et al., 2018a). The controversy remains unresolved on parasympathetic signals restraining tumors instead of fueling them. In our view, the field must disentangle the context-dependent roles of different neural circuits, including sensory nerve-released neuropeptides such as SP and CGRP in various tumor microenvironments. It is still unclear which neural inputs are most critical at distinct tumor phases (initiation, progression, metastasis, or dormancy) and how they dynamically interact. Addressing this requires longitudinal and tissue-specific studies, as well as tools to selectively manipulate one neural subtype at a time.

### Neural influence on immune surveillance

Neuroimmune crosstalk within tumors is undeniably complex. While there is consensus that chronic sympathetic signaling creates an immunosuppressive TME (via TAM polarization, MDSC expansion, T cell dysfunction (Sattler et al., 2025) some findings suggest *nuanced or even opposing effects under different conditions*. For example, β-adrenergic signaling can sometimes enhance certain immune functions (e.g., mobilization of immune progenitors or neutrophils) at lower concentrations, yet potently suppress anti-tumor immunity when stress-related NE levels are high (Qin et al., 2015b). This dose- and context-dependent immune modulation is not fully understood. Similarly, dopamine and serotonin have immunomodulatory roles that can tilt toward immune activation or suppression based on receptor subtypes engaged. Open questions: How do neural signals integrate with checkpoint pathways and cytokine networks in the TME? Might there be situations where neural inputs actually *enhance* immune surveillance (for instance, transient sympathetic activation boosting antigen presentation) rather than suppress it? Advanced *in vivo* imaging and single-cell sequencing of innervated tumors may help map these interactions with high resolution.

### Systemic neuroendocrine circuits in cancer

Most research to date has examined local interactions (nerves within the tumor). However, tumors exist in a whole-body context where the central nervous system (CNS) and endocrine systems respond to malignancy. Psychosocial stress, for example, elevates systemic sympathetic output and cortisol levels (via the HPA axis), which can accelerate tumor progression in animal models (Wang et al., 2025). Yet we lack a clear picture of how brain-mediated factors (stress, circadian rhythms, neurological co-morbidities) influence tumor-immune interactions. Do central neural circuits (like hypothalamic or brainstem pathways) actively “sense” tumors and modulate peripheral immunity accordingly? Conversely, to what extent can tumors send afferent neural signals to the brain to induce behavioral changes (pain, depression, cachexia) that loop back and affect tumor growth (Zhu et al., 2025). These brain-body feedback loops represent a Frontier of cancer neuroscience (Zhu et al., 2025). Unraveling them will require interdisciplinary approaches, integrating neuroimaging, neural circuit manipulation (e.g., vagus nerve stimulation or beta-blockade), and monitoring of systemic physiological changes in cancer patients.

### Limitations of current models

Another major hurdle in the field is the lack of experimental models that fully recapitulate human tumor-nerve-immune circuitry. Standard cell culture systems lack innervation and immune components. Co-culture models using neurons (e.g., dorsal root ganglia co-cultured with tumor organoids) provide valuable insights (Joseph et al., 2025) but still cannot mimic systemic influences (like brain inputs or endocrine signals). *In vivo* murine models have demonstrated the importance of innervation (e.g., surgical or pharmacologic denervation often slows tumor growth (Magnon et al., 2013), yet these models often use young mice without comorbidities or chronic stress, potentially underrepresenting the neuroimmune complexity seen in patients. Moreover, species differences (mouse neurons vs. human) and the short timescale of murine experiments may not capture the chronic neural remodeling of human cancers.

## Conclusion

In conclusion, cancer-neuron interactions represent a Frontier in oncology that bridges tumor biology with neuroscience. Ongoing studies are unraveling the molecular underpinnings of this crosstalk and are beginning to translate those findings into interventions. By viewing tumors not just as masses of mutated cells but as organs enmeshed with nerves, we open the door to *new interventions* that may help stifle tumors at their neurobiological roots. Continued interdisciplinary research will be essential to fully realize therapies that can “cut the lines” of communication between cancer and the nervous system, thereby impeding tumor progression and improving patient survival.
